# Kaliumhydroxid-5 %-Lösung bei aktinischer Keratose: Ein neuer Therapieansatz zur läsionsgerichteten Behandlung

**DOI:** 10.1007/s00105-021-04888-0

**Published:** 2021-08-13

**Authors:** U. Reinhold, J. Bai-Habelski, D. Abeck, R. Denfeld, R. Dominicus, T. Fischer, P. Radny

**Affiliations:** 1 MVZ Dermatologisches Zentrum Bonn GmbH, Friedensplatz 16, 53111 Bonn, Deutschland; 2 Hautzentrum Nymphenburg, Renatastr. 72, 80639 München, Deutschland; 3 Werderstr. 66, 70190 Stuttgart, Deutschland; 4 Hautzentrum Dülmen, Vollenstr. 8, 48249 Dülmen, Deutschland; 5 Haut- und Lasercentrum Potsdam, Kurfürstenstr. 40, 14467 Potsdam, Deutschland; 6 Derma-Study-Center Friedrichshafen GmbH, Charlottenstr. 12/1, 88045 Friedrichshafen, Deutschland

**Keywords:** Medizinprodukt, Läsionsgerichtete Therapie, Komplette Remission, Wirksamkeit, Verträglichkeit, Medical device, Lesion-directed therapy, Complete remission, Efficacy, Safety

## Abstract

**Hintergrund:**

Die aktinische Keratose (AK) ist ein epitheliales Carcinoma in situ der Haut. Aufgrund des Risikos einer malignen Transformation besteht ein frühzeitiger Behandlungsbedarf. Gerade die initiale Therapie sollte neben der Wirksamkeit eine gute Verträglichkeit und Anwenderfreundlichkeit aufweisen. Kaliumhydroxid (KOH)-Lösung ist als keratolytische Behandlungsoption bei hyperkeratotischen Hauterkrankungen, wie z. B. Mollusca contagiosa, bereits etabliert.

**Methodik:**

Wirksamkeit und Verträglichkeit von KOH-5 %-Lösung zur Behandlung der leichten bis moderaten AK wurden in einer prospektiven, einarmigen, multizentrischen Medizinproduktestudie (Treatment of AK with KOH [TAKKOH]) untersucht. Die KOH-Lösung wurde 2‑mal täglich über 14 Tage aufgetragen mit anschließender Behandlungspause von 14 Tagen (≙ 1 Behandlungszyklus) für maximal 3 Behandlungszyklen oder mindestens bis zum Behandlungserfolg. Das primäre Zielkriterium „Behandlungserfolg“ wurde als komplette Remission (CR) aller AK-Läsionen eines Patienten definiert. Sekundäre Zielkriterien beinhalteten die Beurteilung der partiellen Remission (PR), der Anzahl an AK-Läsionen in Remission, die Wirksamkeitsbeurteilung anhand von Schulnoten durch Prüfärzte und Patienten sowie sicherheitsrelevante Endpunkte.

**Ergebnisse:**

Es wurden 73 Patienten in die Studie eingeschlossen. Eine CR wurde von 54,9 % der Patienten erreicht, eine PR von 64,8 % bei einer Reduktion der Gesamtzahl an Läsionen um 69,9 %. Bei 46,6 % der Patienten wurden unerwünschte Ereignisse beobachtet. Diese überwiegend unerwünschten Wirkungen (82,6 %) stellten ausnahmslos transiente und milde lokale Hautreaktionen dar.

**Schlussfolgerung:**

Die Studie liefert Hinweise auf die Wirksamkeit und Sicherheit von KOH-5 %-Lösung zur läsionsgerichteten topischen Therapie der AK.

## Abstract (en)

**Background:**

Actinic keratosis (AK) is an epithelial carcinoma in situ of the skin. There is a need for early treatment due to the risk of malignant transformation. In addition to being effective, the initial therapy in particular should be well tolerated and user-friendly. Potassium hydroxide (KOH) solution is already established as keratolytic treatment option for hyperkeratotic skin diseases such as mollusca contagiosa.

**Materials and methods:**

A prospective single-arm, multicentre medical device study (Treatment of AK with KOH, TAKKOH) was conducted to investigate the efficacy and safety of KOH 5% solution for the treatment of mild to moderate AK. Patients applied KOH solution twice daily for 14 days with a subsequent off-treatment phase of 14 days (≙ one treatment cycle) for a maximum of three treatment cycles or at least until treatment success was achieved. Treatment success, defined as complete remission (CR) of all AK lesions of a patient, was the primary objective. Secondary objectives included the evaluation of partial remission (PR), the number of AK lesions in remission, efficacy assessment by investigators and patients with a 6‑point grading system and several safety parameters.

**Result:**

In all, 73 patients were enrolled in the study. CR was achieved in 54.9% of patients, whereas PR was observed in 64.8% with a 69.9% reduction in lesion numbers. With respect to safety, 46.6% of the patients experienced adverse events. Most of these events (82.6%) were adverse reactions comprising exclusively short-lived and mild local skin reactions.

**Conclusions:**

The study provides an indication of the efficacy and safety of KOH 5% solution for the lesion-directed topical therapy of AK.

Aktinische Keratosen (AK) sind durch die Hyperproliferation atypischer Keratinozyten charakterisiert. Aufgrund des Risikos der Progression oder malignen Transformation wird eine frühzeitige Therapie empfohlen. Da es unter den zahlreichen vorhandenen Therapien bisher keine etablierte Standardtherapie gibt, besteht weiterhin ein Bedarf an wirksamen und sicheren Behandlungsoptionen. In dieser Studie wurde Kaliumhydroxid (KOH), welches als Keratolytikum bereits bei Mollusca contagiosa und anderen viralen Hauterkrankungen eingesetzt wird, erstmals prospektiv in der Therapie der aktinischen Keratose untersucht.

Da für AKs ein beträchtliches Risiko der malignen Transformation besteht, ist eine konsequente Therapie, unabhängig vom Schwere- und Progressionsgrad, unerlässlich [17, 23]. Die Wahl der geeigneten Therapie richtet sich nach patienten-, läsions- und therapieabhängigen Faktoren [12]. Während Feldtherapien bei multiplen, teilweise subklinischen Läsionen angewendet werden, zielen läsionsgerichtete Therapien oftmals auf die physikalische Destruktion einzelner, begrenzter Läsionen ab [12].

Unter den Feldtherapien zählt Diclofenac zu den gut verträglichen, zeigt jedoch nur eine niedrige bis moderate Wirksamkeit [22]. Einige der effektiveren Therapien wie 5‑Fluorouracil (5-FU), Imiquimod oder photodynamische Therapie (PDT) rufen hingegen oftmals andauernde und teilweise schwere Hautreaktionen wie Erythem und Brennen hervor [9].

Läsionsgerichtete Therapien umfassen Verfahren wie Kryochirurgie, Lasertherapie und chirurgische Resektion. Schmerzen, Narbenbildung und Dyspigmentierung zählen hier zu den häufigsten Nebenwirkungen [4].

Derzeit existiert keine etablierte Erstlinientherapie der AK. Daher besteht weiterhin Bedarf an wirksamen, gut verträglichen und anwenderfreundlichen Therapien, insbesondere zur Behandlung leichter bis moderater Formen.

Der Einsatz von KOH als Keratolytikum ist bereits in dermatologischen Indikationen wie Dellwarzen etabliert [1, 8, 16]. Auch andere Therapien wie Imiquimod und 5‑FU/Salicylsäure wurden bereits bei verschiedenen Warzenerkrankungen eingesetzt, bevor ihre Wirksamkeit in der Therapie der AK gezeigt wurde.

Als starke Base führt KOH zu einer unspezifischen chemischen Destruktion atypischer Keratinozyten. Das exzellente Sicherheitsprofil von KOH wurde bereits bei Kindern in der Therapie von Dellwarzen gezeigt [10, 15]. Die vorliegende Studie untersucht erstmals prospektiv die Wirksamkeit und Sicherheit von KOH bei der Behandlung der AK.

## Methodik

### Studiendesign und Patienten

Die vorliegende Studie untersucht als prospektive, multizentrische, einarmige Medizinproduktestudie nach § 23b MPG (Medizinproduktegesetz) die Wirksamkeit und Sicherheit von KOH-5 %-Lösung (Solcera®, InfectoPharm Arzneimittel und Consilium GmbH, Heppenheim, Deutschland) bei immunkompetenten Erwachsenen mit leichter bis moderater AK. Die Studie wurde unter Beachtung der Deklaration von Helsinki, den Prinzipien der guten klinischen Praxis und relevanten nationalen Gesetzen durchgeführt und von der Freiburger Ethik-Kommission GmbH International anerkannt. Das schriftliche Einverständnis für die Studienteilnahme wurde bei jedem Teilnehmer eingeholt.

In die Studie wurden Patienten zwischen 30 und 80 Jahren mit AK-Läsionen Grad I oder II nach Olsen am Körper oder Kopf eingeschlossen. Relevante Ausschlusskriterien waren eine Läsionszahl von über 10 oder ein Durchmesser von über 20 mm einer Einzelläsion.

### Behandlung

**Figure 1:**
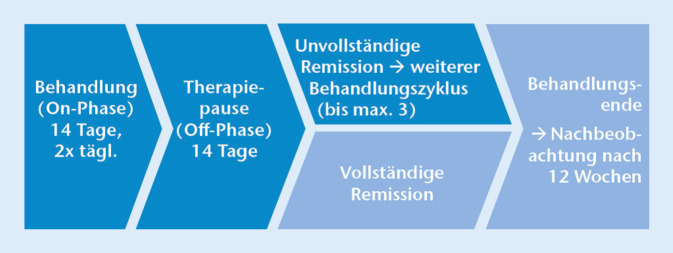

Die KOH-5 %-Lösung wurde von den Patienten mithilfe von produktspezifischen Applikatoren 2‑mal täglich über 14 Tage aufgetragen, gefolgt von einer 14-tägigen Therapiepause (= 28 Tage ≙ 1 Behandlungszyklus). Die erzielte Remission wurde vom Prüfarzt am Ende des ersten Behandlungszyklus beurteilt. Die Behandlungszyklen wurden maximal 3‑mal bis zur Einstellung eines Behandlungserfolges wiederholt. Eine Visite zur Nachbeobachtung („follow-up visit“ [VFU]) wurde 12 Wochen nach der finalen Visite zum Ende der Behandlung (VE), d. h. nach 1, 2 oder 3 Behandlungszyklen, durchgeführt (Abb. 1).

### Primäre und sekundäre Zielkriterien

Als primärer Endpunkt wurde der Behandlungserfolg zu VE, definiert als komplette Remission („complete remission“ [CR]) aller zu Behandlungsbeginn vorhandener und mit der Studienmedikation behandelter Läsionen, festgelegt. Zu den sekundären Zielvariablen zählten die Zeit bis zur CR, die partielle Remission (PR; definiert als CR von mindestens 75 % der Läsionen pro Patient) und die CR auf Läsionsebene, unter anderem differenziert nach Lokalisation.

Darüber hinaus wurde die Anzahl neu auftretender und rezidivierender AK-Läsionen bestimmt. Diese waren definiert als Läsionen, bei denen nach vorangegangener Remission zu VFU ein Rezidiv vorlag. Die Wirksamkeit wurde von Prüfärzten und Patienten mithilfe von Schulnoten bewertet. Außerdem wurde die Therapietreue, definiert als prozentualer Anteil dokumentierter Anwendungen im Verhältnis zur Anzahl vorgesehener Behandlungen, bestimmt.

Die AK-Läsionen wurden durch klinische Untersuchung der Haut und dermatoskopische Bestätigung beurteilt.

### Sicherheit

Zur Bewertung der Sicherheit wurden unerwünschte Ereignisse (UEs) inklusive Art des Ereignisses, Zeitpunkt des Auftretens, Dauer, Schweregrad und Ausgang erhoben und der mögliche Kausalzusammenhang zur Studienmedikation bewertet. Zusätzlich wurde die Verträglichkeit der Behandlung mithilfe von Schulnoten von Prüfärzten und Patienten beurteilt.

### Statistik

Die geplante Fallzahl von 68 Patienten wurde unter Annahme eines Behandlungserfolges von 80 %, einem entsprechenden zweiseitigen 95 %-Konfidenzintervall (KI) mit einer Breite von ±10 % und einer Drop-out-Reserve von 10 % berechnet. Quantitative Daten wurden durch die Berechnung von statistischen Standardparametern analysiert, während qualitative und ordinale Variablen in Form von absoluten und relativen Häufigkeitsverteilungen präsentiert wurden. Das primäre Zielkriterium wurde prospektiv definiert und entsprechend prädefinierter Kriterien ausgewertet. Für die primäre Wirksamkeitsvariable wurde die absolute Häufigkeitsverteilung angegeben und das zweiseitige 95 %-KI nach Clopper-Pearson berechnet. Sekundäre Effektivitätsvariablen wurden deskriptiv entsprechend ihrer Maßeinheit ausgewertet. Primäre und sekundäre Zielkriterien wurden sowohl für den Full-analysis(FA)- als auch für den Per-Protocol(PP)-Datensatz ausgewertet. Sicherheitsendpunkte wurden deskriptiv für den Safety-evaluable(SE)-Datensatz ausgewertet. Für alle UEs wurden absolute und relative Häufigkeitsverteilungen sowie deren Merkmale ausgewertet. Mit Ausnahme der Auswertung zu VFU wurde für alle fehlenden Wirksamkeitsdaten das Last-observation-carried-forward(LOCF)-Prinzip angewendet.

## Ergebnisse

### Patienten

**Figure 2:**
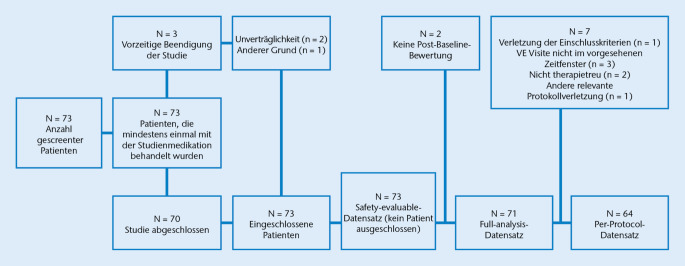

Die Studie wurde in 6 deutschen Studienzentren durchgeführt. Von 73 eingeschlossenen Patienten schlossen 70 die Studie ab, während 3 die Studie vorzeitig beendeten. Die FA-Population, welche zur primären Wirksamkeitsanalyse herangezogen wurde, umfasste 71 Patienten mit mindestens einer Post-Baseline-Bewertung. Die sekundäre Wirksamkeitsanalyse, basierend auf der PP-Population, umfasste 64 Patienten. Die Auswertung der Sicherheitsdaten basierte auf der SE-Population und schloss alle Patienten ein, welche mindestens 1‑mal mit der Studienmedikation behandelt wurden (Abb. 2).

Die Patienten waren im Mittel 66 Jahre alt und hatten mehrheitlich 1 oder 2 Läsionen (42,2 %), während die Anzahl an Läsionen pro Patient im Mittel 3,9 betrug. Die meisten der initial 276 Läsionen waren im Gesicht (52,9 %) oder auf der Kopfhaut (34,4 %) lokalisiert (Tab. 1). Das eingeschlossene Patientenkollektiv war repräsentativ für die Allgemeinpopulation der Patienten mit leichter bis moderater AK.

**Table 1:** 

Variable		Werte
Alter	Mittelwert (SD; Min–Max) [Jahre]	66,0 (8,7; 47–89)
Geschlecht	Männlich Weiblich	39 (54,9 %) 32 (45,1 %)
Hauttyp	I	1 (1,4 %)
	II	58 (81,7 %)
	III	12 (16,9 %)
Zeit seit der erstmaligen Diagnose	Mittelwert (SD) [Jahre]	3,3 (4,1)
Anzahl an AK-Läsionen pro Patient zu Behandlungsbeginn	Mittelwert (SD; Min–Max)	3,9 (2,7; 1–10)
Anzahl und Lokalisation der AK-Läsionen (identifiziert zu Behandlungsbeginn)	Kopfhaut	95 (34,4 %)
	Gesicht	146 (52,9 %)
	Arme und Handrücken	12 (4,4 %)
	Andere	23 (8,3 %)
	Gesamt	276 (100 %)
Größe der AK-Läsionen zu Behandlungsbeginn	Mittelwert (SD; Min–Max) [mm]	8,3 (3,1; 3–18)

*AK* Aktinische Keratose, *SD* „standard deviation“, Standardabweichung

### Wirksamkeit

**Figure 3:**
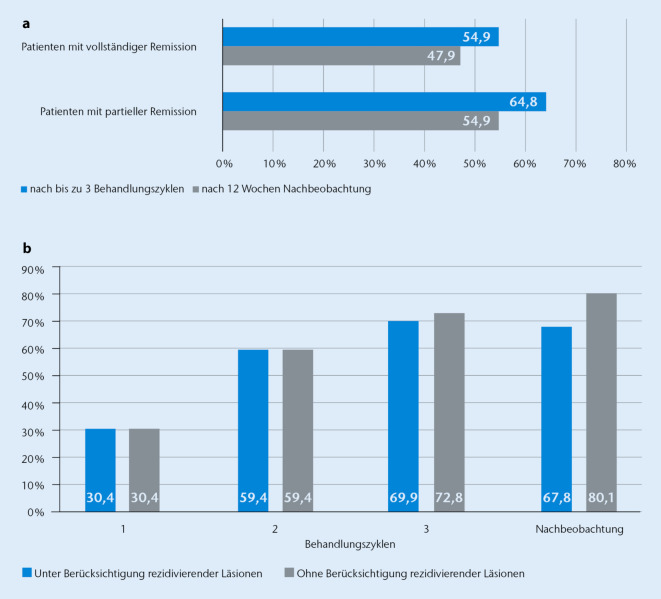

Das primäre Ziel der Studie war die Beurteilung des Behandlungserfolges, definiert als dermatoskopisch bestätigte CR. Nach 1 bis 3 Behandlungszyklen erreichten 54,9 % (39/71; CI 42,7–66,8 %) der Patienten (FA-Population) eine CR. Zu VFU sank die CR-Rate leicht auf 47,9 % (34/71). Die PR-Rate zu VE betrug 64,8 % (46/71) und sank auf 54,9 % (39/71) zu VFU ab (Abb. 3a). Die Zeit bis zur CR betrug im Mittel 58,1 Tage (CI 51,3–64,9; SD 20,9), was ungefähr 2 Behandlungszyklen entspricht.

Vor Behandlungsbeginn wurden insgesamt 276 AK-Läsionen bei 71 Patienten dokumentiert. Von diesen waren 84 (30,4 %) nach dem ersten und 164 (59,4 %) nach dem zweiten Behandlungszyklus in CR. Nach der maximalen Anzahl von 3 Zyklen waren insgesamt 193 (69,9 %) Läsionen vollständig abgeheilt. Obwohl bis zur VFU weitere Läsionen in CR waren, sank, bedingt durch Rezidive, die Anzahl an Läsionen in CR zur VFU leicht auf 187 (67,8 %) (Abb. 3b).

Bis zum Ende des dritten Behandlungszyklus wurden 15 neue Läsionen identifiziert. Bei Berücksichtigung aller vor Behandlungsbeginn und im Studienverlauf identifizierter 291 AK-Läsionen waren 194 Läsionen (66,7 %) zu VFU in CR (3 AK-Läsionen mit fehlender Bewertung). Nach 3 Behandlungszyklen zeigten 8 Läsionen (2,9 %) ein Rezidiv. Zu VFU stieg die Anzahl rezidivierender Läsionen auf 34 (12,3 %) an. Von Behandlungsbeginn bis zu VFU wurde bei 13,4 % (37/276) der Läsionen ein Rezidiv identifiziert. Ohne Berücksichtigung rezidivierender Läsionen waren bis zu VFU 80,1 % der Läsionen zumindest 1‑mal in CR (Abb. 3b).

Initial identifizierte Läsionen befanden sich mehrheitlich im Gesicht (52,9 %) oder auf der Kopfhaut (34,4 %). Für diese Lokalisationen wurden CR-Raten von 74,0 % bzw. 66,3 % zu VE beobachtet. Von 12 Läsionen auf den Armen oder Händen befanden sich zum Behandlungsende 11 (91,7 %) in CR.

**Figure 4:**
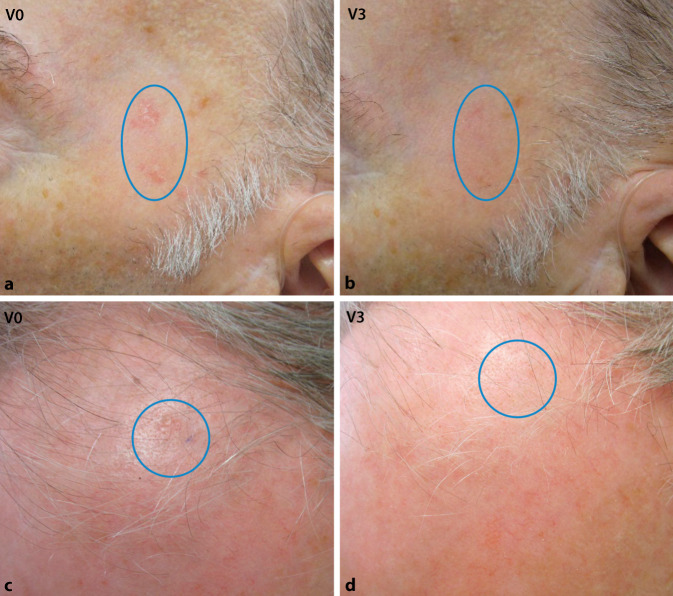

Die Abb. 4 zeigt repräsentative Läsionen im Gesicht und auf der Kopfhaut vor der Behandlung (V0) und nach 3 Behandlungszyklen (V3). Am Ende der Behandlung war die Remission der Läsionen im behandelten Areal deutlich sichtbar (Abb. 4).

**Figure 5:**
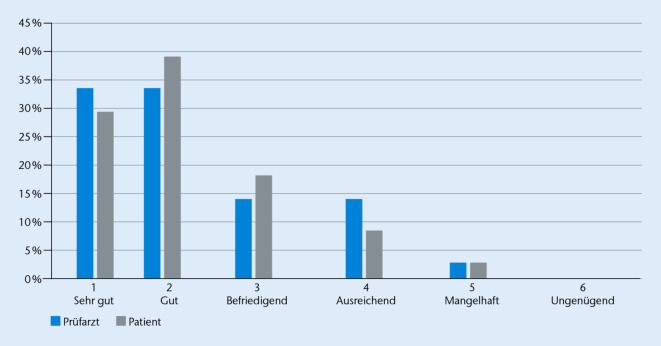

Bei der Bewertung der Wirksamkeit mithilfe von Schulnoten bewertete die Mehrheit der Prüfärzte (48/71; 67,6 %) und Patienten (49/71; 69,0 %) die Wirksamkeit mit „sehr gut“ oder „gut“ (Abb. 5). Die Therapietreue betrug im Mittel 99,4 % (SD 6,5) (Daten nicht gezeigt).

### Sicherheit

Insgesamt wurden 86 UEs bei 46,6 % (34/73) der Patienten im Studienverlauf registriert (SE-Population). Die meisten UEs waren lokale Hautreaktionen (78/86; 90,7 %) in Form von Schmerz, Rötung, Blutung, Juckreiz, Verkrustung und Abschuppung (Tab. 2). Alle der 71 therapieassoziierten UEs (= unerwünschte Wirkungen [UW]) waren lokale Hautreaktionen. Die Mehrzahl der UWs (93,0 %) war leicht und trat vornehmlich im ersten Behandlungszyklus auf (53,8 %). UWs dauerten im Durchschnitt 6,5 Tage bei im Mittel 34,8 Behandlungstagen. Bei keinem der Patienten wurden schwerwiegende UEs beobachtet.

**Table 2:** 

**Sicherheitsprofil auf Ereignisebene**		
**Unerwünschte Ereignisse (UE)**	**Gesamt n (%)**	**86 (100)**
Schwerwiegende unerwünschte Ereignisse (SUE)	–	3 (3,5)
SUE mit Kausalzusammenhang	–	0 (0,0)
Unerwünschte Wirkungen (UW)	–	71 (82,6)
**Unerwünschte Wirkungen (UW)**	**Gesamt n (%)**	**71 (100)**
Lokale Hautreaktionen im Applikationsbereich	Gesamt	71 (100)
Intensität	Leicht Moderat Schwer	66 (93,0) 3 (4,2) 2 (2,8)
Eingeleitete Maßnahme aufgrund der UW	Keine Medikation beendet Medizinische Behandlung Vorzeitige Beendigung der Studie	69 (97,2) 2 (2,8) 0 (0,0) 0 (0,0)
Ausgang	Wiederhergestellt Andauernd Wiederhergestellt mit bleibendem Schaden Unbekannt	70 (98,6) 0 (0,0) 0 (0,0) 1 (1,4)
Dauer der lokalen Hautreaktion	Mittelwert (SD; Min–Max)	6,5 (5,7; 1–18) Tage
**Sicherheitsprofil auf Patientenebene**		
**Patienten mit UEs**^*a*^		
**Gesamt n (%)**		**34 (46,6)**
Apikoektomie		1 (1,4)
Vorhofflimmern		1 (1,4)
Tiefe Venenthrombose		1 (1,4)
Gastrointestinale Infektion		1 (1,4)
Kopfschmerzen		1 (1,4)
Nasopharyngitis		1 (1,4)
Pharyngitis		1 (1,4)
Lungenembolie		1 (1,4)
Zahnschmerzen		1 (1,4)
**Patienten mit UWs/lokalen Hautreaktionen**		
**Gesamt n (%)**		**32 (43,8)**
Schmerz		27 (37,0)
Rötung		5 (6,8)
Blutung		3 (4,1)
Juckreiz		3 (4,1)
Verkrustung		3 (4,1)
Abschuppung		1 (1,4)

*SD* „standard deviation“, Standardabweichung

^a^UEs, bei denen ein Kausalzusammenhang zur Studienmedikation vermutet wurde, sind nachfolgend als UWs aufgeführt

Für die Bewertung der UWs, der Intensität, der eingeleiteten Maßnahmen und des Ausgangs fehlten die Daten für 6 UEs. Für die Bewertung der Dauer der lokalen Hautreaktion fehlten die Daten für 2 UWs

Siebzig von 73 Patienten schlossen die Studie ab. Zwei Patienten (2,7 %) beendeten die Studie aufgrund von Unverträglichkeit (Blutung und Verkrustung im Behandlungsareal) und 1 Patient (1,4 %) aufgrund von Schwierigkeiten mit der Anwendung des Produktes vorzeitig.

**Figure 6:**
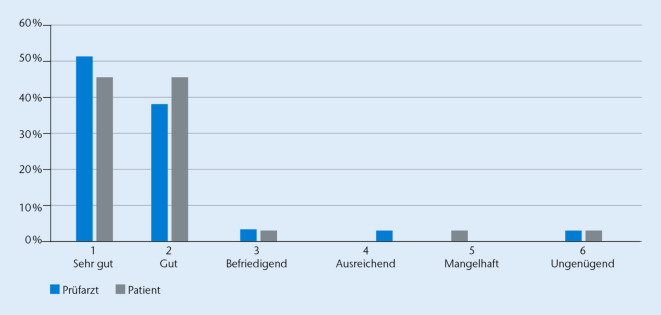

Die Mehrzahl der Prüfärzte (90,5 %; 66/73) und Patienten (93,2 %; 68/73) beurteilten die Verträglichkeit der KOH-5 %-Lösung als „sehr gut“ oder „gut“ (Abb. 6).

## Diskussion

Obwohl bereits einige Therapien zur Behandlung der AK verfügbar sind, besteht weiterhin Bedarf an neuen Therapien mit guter Wirksamkeit, Verträglichkeit und Patientenakzeptanz. In der vorliegenden Studie wurde erstmals KOH-5 %-Lösung als neue topische Behandlungsoption zur läsionsgerichteten Therapie der AK prospektiv untersucht.

### Bewertung der Wirksamkeit

Die CR-Rate von 54,9 % auf Patientenebene und die Reduktion der Läsionszahl um 69,9 % nach 3 Behandlungszyklen deutet auf eine gute Wirksamkeit der neuen Therapie hin. Die deutliche Zunahme des Anteils an Läsionen in Remission von 30,4 % nach dem ersten Behandlungszyklus auf 59,4 % nach dem zweiten und auf bis zu 69,9 % nach dem dritten Zyklus bestätigt die Eignung des Therapieschemas, welches bis zu 3 Zyklen erlaubt.

Die erreichte CR-Rate von 54,9 % auf Patientenebene scheint vergleichbar mit anderen effektiven Therapien wie Imiquimod 5 % oder 5‑FU (mit oder ohne Salicylsäure) zu sein, für welche Remissionsraten zwischen 40 und 60 % berichtet wurden. Demgegenüber liegen die CR-Raten bei verschiedenen Formen der PDT normalerweise bei über 60 %. Für andere etablierte Therapien wie Diclofenac und Imiquimod 3,75 % wurden geringfügig niedrigere Remissionsraten zwischen 30 und 50 % gezeigt [18, 20, 22].

Die Vergleichbarkeit der CR-Raten auf Patientenebene ist jedoch limitiert, da in der TAKKOH-Studie Patienten mit einer vergleichsweise niedrigen durchschnittlichen Läsionszahl eingeschlossen waren, was die Wahrscheinlichkeit für eine CR im Verhältnis zu anderen Studien erhöht.

Die Beurteilung der Wirksamkeit auf Läsionsebene erlaubt einen verlässlicheren Vergleich von Studienergebnissen. Hier wurde für die Behandlung mit KOH-5 %-Lösung eine Reduktion der Anzahl an Läsionen um 69,9 % gezeigt. Im Vergleich wurden für andere, etablierte Therapeutika wie 5‑FU (mit oder ohne Salicylsäure), PDT, Imiquimod und Kryochirurgie höhere maximale Werte für die Reduktion der Läsionszahl von 80 % und mehr beobachtet. Dabei wurden jedoch insgesamt sehr breite Intervalle der Wirksamkeitsraten auf Läsionsebene zwischen 40 und 100 % berichtet, was auch hier den Vergleich der Therapien erschwert [5] und darauf hindeutet, dass neben der initialen Läsionszahl auch weitere Faktoren wie Schweregrad und Erkrankungsstadium einen Einfluss auf die erzielte Wirksamkeit haben und Studien mit unterschiedlichen Patientenkollektiven somit nur bedingt vergleichbar sind.

Eine Subgruppenanalyse der Wirksamkeit an schwer zu behandelnden Lokalisationen (Hände, Unterarme) auf Läsionsebene ergab für KOH eine CR-Rate von über 90 % (Läsionszahl *n* = 12). In einer nichtinterventionellen Studie wurde eine vergleichbare Läsionsreduktion von 92 % an Händen und Armen unter einer Behandlung mit 5‑FU/Salicylsäure beobachtet [14]. Diese Daten legen nahe, dass der sowohl durch Salicylsäure als auch durch KOH hervorgerufene keratolytische Effekt hohe Abheilungsraten an Händen und Armen begünstigt, da es sich hierbei oftmals um hyperkeratotische Läsionen handelt [4].

### Bewertung der Sicherheit

Abgesehen von der potenziell durch KOH hervorgerufenen Reizung oder Verätzung bestehen laut Angaben aus Datenbanken keine Hinweise auf toxikologische Eigenschaften in der Langzeitanwendung [2, 21]. Als Keratolytikum wird KOH seit Jahrzehnten regelmäßig in der Diagnose von Pilzinfektionen eingesetzt [6]. Die Sicherheit der topischen Behandlung mit KOH ist für zahlreiche dermatologische Erkrankungen bei Erwachsenen und Kindern belegt [6, 10, 15].

In der vorliegenden Studie wurde ein gutes Sicherheitsprofil in der Indikation der AK bestätigt. Die Gesamtrate an Patienten mit UE (46,6 %) erscheint ausgesprochen niedrig im Vergleich mit UE-Raten von 85 % und höher, wie sie für Therapien wie Imiquimod, 5‑FU und PDT berichtet wurden [9]. Zudem war die Mehrheit der in dieser Studie aufgetretenen UW leicht, reversibel und von kurzer Dauer. Im Gegensatz dazu wurde bei anderen topischen Therapien ein relevanter Anteil an moderaten bis schweren, teilweise über mehr als 2 Wochen persistierenden Hautreaktionen beobachtet [3, 9].

Insgesamt bestätigen die Daten eine gute bis sehr gute Verträglichkeit der KOH-5 %-Lösung, weshalb in Zusammenschau mit der erzielten Wirksamkeit auf ein positives Nutzen-Risiko-Verhältnis geschlossen werden kann. Die Behandlung mit KOH 5 % stellt somit eine vielversprechende Therapieoption für die Initiierung einer AK-Therapie dar, insbesondere bei Patienten mit nur wenigen Läsionen, um potenziell nebenwirkungsreichere Therapien vorerst zu vermeiden.

### Limitationen

Bedingt durch das Design, weist diese Studie einige Limitationen auf. Die Nachbeobachtungszeit von 12 Wochen erlaubt keine umfassende Bewertung rezidivierender Läsionen sowie des Potenzials der malignen Transformation. Um die Akzeptanz der Intervention durch die Studienteilnehmer zu erhöhen, wurden zudem keine Probebiopsien durchgeführt. Dies kann im Einzelfall die Beurteilung der Läsionsabheilung erschweren. Das einarmige Studiendesign lässt keinen direkten Vergleich mit Placebo oder anderen AK-Therapien zu, wodurch die Aussagekraft der vorliegenden Arbeit begrenzt ist.

Der indirekte Vergleich mit anderen Studien wird durch die Heterogenität der Patientenpopulationen und Methodiken limitiert. Insbesondere die niedrige Anzahl von im Mittel 3,9 Läsionen pro Patient in dieser Studie könnte die Ergebnisse zur CR im Vergleich zu Studien mit höheren Läsionszahlen pro Patient verzerren.

Jedoch ist aufgrund der Progressivität der Erkrankung von einer niedrigen Spontanheilungsrate der AK-Läsionen auszugehen, was durch konsistent niedrige Placebowirksamkeiten in einer Vielzahl von Studien gestützt wird [22]. Daher ergeben die berichteten niedrigen Placebowirksamkeiten (CR unter 10 % und Reduktion der Läsionszahl zwischen 20 und 25 % [7, 11, 13, 19, 22]) im Vergleich zu einer CR-Rate von 54,9 % und einer Reduktion der Läsionszahl um 69,9 % in dieser Studie deutliche Hinweise auf eine gute Wirksamkeit von KOH-5 %-Lösung. In einigen Studien wird zudem von einem relevanten Vehikeleffekt berichtet, was in diesem Fall zur Unterschätzung der Überlegenheit von KOH über ein „echtes“ Placebo führen würde.

## Ausblick

Unter Berücksichtigung der Limitationen des einarmigen Studiendesigns deuten die Ergebnisse darauf hin, dass KOH-5 %-Lösung eine wirksame und sichere läsionsgerichtete Therapieoption für die Behandlung der AK darstellt. Aufgrund ihres vorteilhaften Risikoprofils eignet sich KOH insbesondere für die initiale Therapie weniger Läsionen, kann aber möglicherweise auch in der sequenziellen Therapie in Kombination mit feldgerichteten Therapien wirksam eingesetzt werden. KOH-5 %-Lösung stellt somit eine wertvolle neue Behandlungsoption dar. Weitere Studien in einem kontrollierten Setting sind wünschenswert.

## Fazit für die Praxis

- Nach topischer Therapie mit Kaliumhydroxid(KOH)-5 %-Lösung reduzierte sich die Anzahl an aktinischen Keratosen um 70 % im Vergleich zum Ausgangswert.
- Die Therapie kann einfach vom Patienten selbst durchgeführt werden und zeichnet sich durch eine gute bis sehr gute Verträglichkeit aus.
- Für die Zukunft sind Daten aus kontrollierten Studien insbesondere im Vergleich mit etablierten Therapien wünschenswert, um die Wirksamkeit von KOH-5 %-Lösung bei aktinischer Keratose zu bestätigen.
